# Evaluation of the mobile application “Descomplicando a Amamentação” by family members of newborns *

**DOI:** 10.1590/1518-8345.6883.4086

**Published:** 2023-12-04

**Authors:** Ingrid Lucchese, Fernanda Garcia Bezerra Góes, Andressa Neto Souza, Aline Cerqueira Santos Santana da Silva, Liliane Faria da Silva, Iasmym Alves de Andrade Soares

**Affiliations:** 1 Universidade Federal Fluminense, Instituto de Humanidades e Saúde, Rio das Ostras, RJ, Brasil.; 2 Becaria del Conselho Nacional de Desenvolvimento Científico e Tecnológico (CNPq), Brasil.; 3 Universidade Federal do Estado do Rio de Janeiro, Rio de Janeiro, RJ, Brasil.; 4 Universidade Federal do Estado do Rio de Janeiro, Escola de Enfermagem Alfredo Pinto, Rio de Janeiro, RJ, Brasil.; 5 Becaria de la Coordenação de Aperfeiçoamento de Pessoal de Nível Superior (CAPES), Brasil.; 6 Universidade Federal Fluminense, Escola de Enfermagem Aurora de Afonso Costa, Niterói, RJ, Brasil.; 7 Becaria de la Fundação de Amparo à Pesquisa do Estado do Rio de Janeiro (FAPERJ), Brasil.

**Keywords:** Child Health, Breast Feeding, Mobile Applications, Educational Technology, Health Education, Family, Salud Infantil, Lactancia Materna, Aplicaciones Móviles, Tecnología Educacional, Educación en Salud, Familia, Saúde da Criança, Aleitamento Materno, Aplicativos Móveis, Tecnologia Educacional, Educação em Saúde, Família

## Abstract

to evaluate the semantics, appearance and usability of the mobile application “*Descomplicando a Amamentação*” for family members of newborns.

applied methodological study, developed with 20 family members of newborns users of educational technology, including pregnant women, postpartum women and family members of newborns. An evaluation instrument containing questions about semantics and appearance was applied, in addition to the System Usability Scale to evaluate usability. In data analysis, the Agreement Index was used, with a cutoff point of 0.8 (80%).

when evaluating semantics and appearance, the application achieved a Global Agreement Index of 0.99 (99%), varying from 0.95 (95%) to 1.0 (100%) between the items evaluated. In usability, a global average of 93 was achieved, presenting the best usability achievable in all system characteristics.

the technology was considered understandable, relevant, and efficient, as well as easy to use and low inconsistency with high user satisfaction, demonstrating excellent potential for families.

## Abstract (es)

evaluar la semántica, la apariencia y la usabilidad de la aplicación móvil *Descomplicando a Amamentação* por familiares de recién nacidos.

estudio metodológico aplicado, desarrollado con 20 familiares de recién nacidos ususrios de la tecnología educativa, siendo embarazadas, puérperas y familiares de recién nacidos. Se aplicó un instrumento evaluativo conteniendo preguntas sobre la semántica y la apariencia, además de la *System Usability Scale* para evaluación de la usabilidad. En el análisis de los datos se utilizó el Índice de Concordancia, con un punto de corte igual a 0,8 (80%)

en la evaluación de la semántica y de la apariencia, la aplicación alcanzó un Índice de Concordancia Global de 0,99 (99%), presentando una variación de 0,95 (95%) a 1,0 (100%) entre los ítems evaluados. En la usabilidad, se alcanzó una media global de 93, presentando la mejor usabilidad alcanzable en todas las características del sistema.

se consideró la tecnología comprensible, relevante, pertinente y eficiente, además de fácil utilización y baja inconsistencia con alta satisfacción de los usuarios, demostrando excelente potencial de uso por las familias

## Abstract (pt)

avaliar a semântica, a aparência e a usabilidade do aplicativo móvel “Descomplicando a Amamentação” por familiares de recém-nascidos.

estudo metodológico aplicado, desenvolvido com 20 membros familiares de recém-nascidos usuários de tecnologia educacional, incluindo gestantes, puérperas e familiares de recémnascidos. Aplicou-se um instrumento avaliativo contendo questões sobre a semântica e a aparência, além da *System Usability Scale* para avaliação da usabilidade. Na análise dos dados, utilizou-se o Índice de Concordância, com ponto de corte igual a 0,8 (80%).

na avaliação da semântica e da aparência, o aplicativo alcançou um Índice de Concordância Global de 0,99 (99%), apresentando variação de 0,95 (95%) a 1,0 (100%) entre os itens avaliados. Na usabilidade alcançouse média global de 93, indicando melhor grau alcançável em todas as características do sistema.

a tecnologia é compreensível, relevante, pertinente e eficiente, além da fácil utilização e baixa inconsistência com elevada satisfação pela amostra investigada, mostrando excelente potencial de uso pelas famílias.

**Destacados:**

**(1)** The study evaluated a mobile application about breastfeeding.

**(2)** Twenty family members of newborns participated in the assessment.

**(3)** The evaluation of semantics, appearance and usability was excellent.

**(4)** The app evaluated can be applied to family health education.

**(5)** *“Descomplicando a Amamentação”* can be accessed at any time and place.

## Introduction

The World Health Organization (WHO), the Brazilian Ministry of Health (MS) and the American Academy of Pediatrics (AAP) recommend exclusive breastfeeding (EBF) in the first six months of life – in which the child should be deprived of consuming other liquid and solid foods – and supplemented until the age of two or more ^(^ 1 ^)^. Breast milk provides protection against different infections, diarrhea, respiratory diseases and allergies and also promotes bonding between mother and child. It is the most suitable and complete food for babies, as its components are essential for child growth and development ^(^ 2 ^-^ 3 ^)^. 

Despite global advances, such as the implementation of public policies and programs to encourage this practice, rates of early initiation, duration and exclusivity have not yet reached desirable levels ^(^ 1 ^)^. Less than 35% of mothers on the planet breastfeed their children exclusively for the recommended period. In Brazil and several other countries, the goals recommended by the WHO are still far from being achieved ^(^ 4 ^)^. It is known that there are numerous factors that influence the initiation and continuation of breastfeeding and some of the main predictors are maternal intention to breastfeed, lack of knowledge about improving lactation and little confidence in having skills to breastfeed ^(^ 5 ^)^. 

Educational health actions can contribute to minimizing possible obstacles and encouraging breastfeeding, in order to increase these rates through effective communication between families and professionals, especially with the help of technologies that enable the construction of collective knowledge that leads to changes in attitudes, through dialogue and critical reflection. The use of technologies in this area favors the expansion of access to information on the subject, assistance to mothers and family members in matters regarding the practice of breastfeeding and the increase in the prevalence and duration of breastfeeding ^(^ 6 ^)^, enabling the application of methodologies in health learning activities. 

It is worth highlighting that research showed that women mainly use the internet and mobile devices to search for information during the perinatal period and, commonly, download health-related applications on their smartphones ^(^ 7 ^)^. Worldwide, mobile applications in healthcare, also called mobile health ( *mHealth*), are increasingly used to complement healthcare interventions, being used, for example, to interact with customers or share secure information, in order to improve the quality of care and the empowerment of family members ^(^ 8 ^)^. 

According to the 33 ^rd^ Annual Survey on the Use of Information Technologies by Fundação Getúlio Vargas, 447 million digital devices were counted in Brazil (computer, notebook, tablet and smartphone) in June 2022, that is, more than two devices per inhabitant in the country. Of these devices, the smartphone is the most used for banking transactions, purchases and use of social media ^(^ 9 ^)^. Due to the rapid dissemination of mobile devices in society, especially smartphones, it is believed that the use of educational health technologies through these devices is a promising strategy for nurses to act in a more dynamic and innovative way as health educators, given the possibility of dissemination of health information in a comprehensive manner to different audiences ^(^ 10 ^)^. 

Due to growing concerns about the quality of applications that deal with infant feeding, it is essential to test scientific research on smartphone applications that provide reliable information about breastfeeding ^(^ 5 ^)^. Thus, recently, an application for mobile devices, entitled “ *Descomplicando a Amamentação*”, was developed by nurses and validated by experts as an educational technology about breastfeeding, with the potential to help mothers and families about this practice in a didactic, interactive way, free and independent. The technology is made up of an educational booklet, in addition to other tabs that cover information about the main problems with breasts and breastfeeding during COVID-19, and also a quiz about the main doubts regarding the topic ^(^ 11 ^)^. 

The objective of evaluating a new technology is to contribute to the improvement of its functions and ensure that the contents and functions are appropriate to its intentions. However, to ensure the development and use of an application is valued, it is necessary not only to validate it with professionals with expertise in the area, but also to evaluate its semantics, appearance and usability with the intended target audience ^(^ 12 ^)^. Therefore, the inclusion of the end user in the evaluation process is essential, as only the real recipients can directly point out what is missing for them to identify with the educational material, thus guaranteeing the best refinement of the technology ^(^ 13 ^-^ 14 ^)^. 

In the process of evaluating educational technologies, the main types used are semantic and appearance evaluation, which seeks to evaluate whether the technology is understandable for the intended audience, being an essential step for reformulating language and images and thus enabling easy-to-understand material for the reality of each audience ^(^ 15 ^)^. In addition to these, the level of usability of a technology can also be measured, relating to the assessment of the software’s ability to be operated by an individual, as well as the ability of users to be able to perform specific tasks with ease when interacting with such a system ^(^ 16 ^-^ 17 ^)^. Therefore, usability assessment allows technological products to be improved so that the user achieves their interaction objectives in an uncomplicated manner ^(^ 18 ^)^. However, investigations into the evaluation of educational health technologies related to breastfeeding among the target audience are still scarce in Brazil, which justifies this study. 

Given the potential use of the mobile application “ *Descomplicando a Amamentação*” as an educational health technology that facilitates decision-making by pregnant women, postpartum women and family caregivers in adhering to and maintaining breastfeeding, by expanding their knowledge in a practical and accessible way, it is necessary to validate it with the target audience. Therefore, the objective of the study was to evaluate the semantics, appearance and usability of the “ *Descomplicando a Amamentação*” mobile application for family members of newborns. 

## Method

### Type of study and period

Applied methodological study that evaluated an educational health technology ^(^ 19 ^)^, in the format of an application for mobile devices between November/2022 and January/2023. 

### Study location

The study was carried out in the maternity ward of a municipal hospital located in the coastal lowlands of the state of Rio de Janeiro, Brazil. This low and medium risk maternity hospital is a reference in assistance to parturient women residing in the municipality and surrounding cities. In the unit, women in labor are taken to the delivery room or surgical center and, after birth, the baby is taken to rooming-in.

### Population and selection criteria

The population was made up of pregnant women, postpartum women and family members of newborns who were born in the research scenario. The following inclusion criteria were applied: pregnant women over the age of 18 who were hospitalized in rooming-in and postpartum women and family members over the age of 18 whose newborns were in good health and hospitalized in the research setting. Pregnant women, postpartum women and/or family members who presented any impairment in their health status that made it difficult to evaluate the application were excluded.

### Sample

The sample was non-probabilistic and for convenience, composed of 20 end users of educational technology, in accordance with scientific literature that suggests six to twenty participants for each group of evaluators in studies of this nature ^(^ 20 ^)^. Family members present at the institution during the data collection period were chosen according to the selection criteria and their availability to participate in the study until the sample number was reached. 

### Study variables

The first part of the assessment instrument was composed of closed questions that allowed the characterization of the participants, namely: age, sex, education, profession and degree of kinship with the newborn. The second part included questions specifically related to the object of study, aimed at evaluating semantics, appearance and usability.

### Instruments used to collect information

The application’s evaluation instrument was composed of questions about semantics (09 questions) and appearance (07 questions), which were scored using a Likert scale, ranging from 1 to 4 points, namely: “I strongly disagree” (1 point), “I disagree a little” (2 points), “I agree a little” (3 points) and “I agree a lot” (4 points) ^(^ 11 ^)^. Sequentially, the usability of the application was evaluated using the System Usability Scale (SUS), developed by John Brooke in 1986 ^(^ 21 ^)^, consisting of ten items and which also uses a Likert scale, but with a range of 1 to 5 points, namely: “I disagree a lot” (1 point), “I disagree a little” (2 points), “I neither agree nor disagree” (3 points), “I agree a little” (4 points) and “I agree a lot” (5 points) ^(^ 16 ^)^. At the end of the evaluation instrument, a space was made available for suggestions and comments aimed at improving educational health technology. 

### Data collection

Pregnant women, postpartum women and family members were approached personally during hospitalization in the rooming-in wards, being invited to participate in the research and upon acceptance, they evaluated on a tablet the version of the application “ *Descomplicando a Amamentação*”, which is available for free download, on the Google Play Store platform for the Android operating system and is registered with the National Institute of Industrial Property (INPI), #BR512021001467-7. After using the application, the semantics, appearance and usability in a printed form were evaluated. It is noteworthy that each participant took an average of 20 minutes to evaluate the application and respond to the evaluation instrument. 

### Data processing and analysis

The data relating to the evaluation of the application were analyzed using the Agreement Index (AI), calculated item by item, based on the sum of the two responses that denoted a more positive appreciation of the application (agreement) divided by the total number of responses. In the items referring to semantics and appearance and in the odd answers of the SUS scale, the most positive answers were “somewhat agree” and “very much agree” and in the even numbers of the SUS scale “very much disagree” and “somewhat disagree”, as they are questions with items inverted.

The global AI for the semantic and appearance assessment was defined based on the average of the 16 individual items related to these topics. The calculation of the general usability score of the SUS scale was generated by summing the particular collaboration of each item. For odd items, one point was subtracted from the value given to that answer. For even items, the calculation was made by subtracting the value given to the answer from the total of five points. To calculate the total score, the values achieved from the even and odd items were added together and multiplied by 2.5. Therefore, the total usability score varies between 0 and 100 points ^(^ 16 ^)^. 

AIs that achieved a score equal to or greater than 0.8 (80%) ^(^ 22 ^)^ were considered valid. In addition, a score in the general score of the SUS scale between 0 and 25 would indicate a worse usability level achievable; from 26 to 39 bad; 40 to 52 acceptable; 53 to 74 good; 75 to 85 excellent and 86 to 100 best achievable ^(^ 23 ^)^, therefore, items that were not considered acceptable would be reviewed. 

Using the SUS scale, it is possible to evaluate the five main characteristics for the usability of an application: 1) ease of knowing the system - items 3, 4, 7 and 10; 2) system efficiency - items 5, 6 and 8; 3) inconsistencies - item 6; 4) ease of memorization - item 2; 5) user satisfaction - items 1, 4 and 9. To score individual usability characteristics, the responses obtained from each participant per item were multiplied by 25, in order to obtain the range of possible values from 0 to 100. Afterwards, the general average was calculated between the scores obtained for each question and then the average between the items referring to the characteristics ^(^ 23 ^-^ 24 ^)^. 

It is noteworthy that only one round of evaluation of the target audience was necessary, given the high AI obtained in the evaluation of all items referring to semantics, appearance and usability, in addition to the excellent values in the global AI and general score of the SUS scale. Therefore, it was not necessary to adapt the educational material after this evaluation process, as only one participant made a suggestion for modification regarding the rotation of breasts for breastfeeding, which will be considered later in a new version of the application.

### Ethical aspects

The study was submitted to the Research Ethics Committee (CEP) of the Fluminense Federal University for consideration and approval (#4.051.040, CAAE: 29155419.9.0000.5243), in accordance with Resolution 466 of 2012 of the National Health Council, which provides for the ethical aspects of research involving human beings. Data were collected by personally signing the Informed Consent Form (ICF), guaranteeing confidentiality of all information collected.

## Results

Twenty people (pregnant women, postpartum women and family members of newborns) participated in the evaluation process, with an average age of 27.6 years, ranging between 18 and 46 years; 17 (85%) were female and three (15%) were male; 11 (55%) with secondary education, six (30%) with higher education and three (15%) with primary education; nine (45%) housewives and the remaining 11 (55%) distributed in different professions; 16 (80%) were the mothers of the newborns, three (15%) were the fathers and one (5%) was a cousin. Table 1 presents the target audience’s evaluation regarding semantics and appearance, according to the AI per item and overall. 

**Table 1 -:** Assessment of the target audience regarding semantics and appearance (n=20). Rio das Ostras, RJ, Brazil, 2023

Regarding semantics			
Item	I disagree a lot/ I disagree a little	I agree a lot/ I agree a little	Item AI*
The language used in the application is easy to understand	0	20	1.0
The information is clear	0	20	1.0
The app makes it easier to learn how to breastfeed	1	19	0.95
Invites and/or attracts you to changes in newborn breastfeeding habits	0	20	1.0
The quiz clearly addresses the main breastfeeding issues	0	20	1.0
The quiz is attractive	1	19	0.95
The app provides help in a positive way	0	20	1.0
The app made you think about breastfeeding	0	20	1.0
The app motivated you to change your breastfeeding habits	1	19	0.95
**Regarding appearance**			
**Item**	**I disagree a lot/I** **disagree a little**	**I agree a lot/ I agree** **a little**	Item AI*
The letters are in the appropriate size	0	20	1
The app is attractive	0	20	1
The images are easy to understand	0	20	1
The colors are appropriate	0	20	1
The app looks organized	0	20	1
The application is easy to use	0	20	1
All screens keep common application menus and functions accessible	0	20	1
AI* Global Average = 0.99			

*AI = Agreement Index

The average AI for all items was greater than 0.8 (80%), both for semantics and appearance, indicating that the application presented a satisfactory evaluation, reaching an overall average value of 0.99 (99%). In the evaluative items, there was a variation from 0.95 (95%) to 1.0 (100%). It is noteworthy that in appearance all items had AI of 1.0 (100%). Table 2 presents the target audience’s evaluation regarding usability, according to the AI per item and the general score of the SUS scale. 

**Table 2 -:** Assessment of the target audience regarding usability using the System Usability Scale (SUS) (n=20). Rio das Ostras, RJ, Brazil, 2023

Item	I disagree a lot/I disagree a little	I do not agree nor disagree	I agree a lot/ I agree a little	Item AI*
1. I would use this app frequently	1	3	16	0.8
2. I found the application unnecessarily complex	18	0	2	0.9
3. I found the app easy to use	0	0	20	1.0
4. I think I would need technical support to be able to use this application	20	0	0	1.0
5. I thought the application’s various functions were well integrated	0	0	20	1.0
6. I thought there was a lot of inconsistency in this app.	18	1	1	0.9
7. I would imagine most people would learn to use	1	4	16	0.8
8. I found the application too cumbersome to use	19	1	0	0.95
9. I felt very confident using the app	1	1	18	0.9
10. I needed to learn a number of things before I could continue using the application	19	1	0	0.95
AI* Global Average = 0.92				
**General SUS Scale Score = 93**

*AI = Agreement Index

Regarding usability assessment using the SUS scale, it was identified that the application presented an overall average SUS score of 93, with scores ranging from 72.5 to 100.0; classifying itself with a better achievable degree of usability. Furthermore, the AI of all items was equal to or greater than 0.8 (80%), ranging from 0.8 (80%) to 1.0 (100%), with a mean of 0.92 (92%), which again indicates a satisfactory assessment. Therefore, all items about semantics, appearance and usability of the “ *Descomplicando a Amamentação*” application presented excellent scores in the evaluation of family members of newborns. 

Furthermore, the items on the SUS scale also have specific usability characteristics with relevant meanings, as a way of evaluating the quality components of a software ( Table 3). 

The results indicated a range of 92.9 to 96.3 between the usability characteristics of the application, indicating that they all achieved rates classified as best achievable, being, therefore, an educational technology that presents high ease of knowledge and memorization of the system, as well such as high satisfaction and efficiency, as well as low inconsistency.

It should be noted that regarding semantics, one participant suggested reviewing the correct alternative or changing the wording of the tenth question of the quiz: “Is it necessary to alternate both breasts when breastfeeding?”, as the answer is “false”, but one must change breasts after the first breast is empty, switching from one feeding to another. This suggestion will be considered in a new version of the application with a new wording of the question to: “Is it always necessary to alternate both breasts when breastfeeding during the same feeding?”. There were no suggestions for appearance and usability.

**Tabla 3 -:** Evaluación del público objetivo respecto a las características de usabilidad (n=20). Rio das Ostras, RJ, Brasil, 2023

Usability characteristics	Average of items across participants	Overall average	Meaning
Easy to understand the system	I3 (98.8)	95.3	Easy-to-use system when used for the first time
	I4 (97.5)		
	I7 (87.5)		
	I10 (97.5)		
System efficiency	I5 (96.3)	95.5	Speed in carrying out established tasks
	I6 (96.3)		
	I8 (93.8)		
Inconsistencies	I6 (96.3)	96.3	Absence of errors
Ease of memorization	I2 (96.3)	96.3	Easy to use system even after a long period of non-use
User satisfaction	I1 (87.5)	92.9	Nice design
	I4 (97.5)		
	I9 (93.8)		

## Discussion

The objective of the study to evaluate the semantics, appearance and usability of the mobile application “ *Descomplicando a Amamentação*” by family members of newborns was satisfactorily achieved, through AI on semantics and appearance, both per item and overall, above the scores desirable, in addition to a better achievable level of usability using the SUS scale, which demonstrates excellent potential for use by families. It is noteworthy that this type of health technology has shown itself to be promising, as it tends to contribute positively to promoting breastfeeding, increasing the prevalence rates of this practice and reducing the introduction of other foods into the baby’s diet before the sixth month. of life ^(^ 6 ^)^. 

The evaluation of educational materials is an essential step in making them available and this process with the intended target audience makes it possible to identify and improve the aspects that involve them ^(^ 25 ^)^, especially with regard to their understanding and relevance, contributing, thus, with research and care practice in the area of nursing, as this process provides greater reliability and credibility to educational technologies ^(^ 26 ^-^ 27 ^)^. Therefore, as mobile applications have become a popular platform used to support breastfeeding, they must also be evaluated by the target audience to ensure their usability, as occurred in a study carried out in Thailand ^(^ 28 ^)^. This type of evaluation therefore emerges as an essential component for the provision of a reliable, relevant and trustworthy technological product from the perspective of the users themselves. 

The results of this research were similar to other studies on educational technologies on breastfeeding in Brazil, such as an educational video validated regarding its functionality, usability, efficiency, audiovisual technique, environment, procedure, objectives, organization, video style, appearance and motivation, which obtained an overall rating of 100% by the target audience, and can be used to encourage this practice among families ^(^ 27 ^)^. Another investigation that sought to evaluate educational material in the format of a comic book about breastfeeding with children between seven and nine years of age obtained an agreement rate of 95.6% in the evaluation by the target audience, thus revealing itself to be a valid and reliable instrument to be used with schoolchildren in order to promote the culture of breastfeeding ^(^ 29 ^)^. 

Regarding the evaluation of applications related to breastfeeding in Brazil, a study that developed and validated a technological product of this nature for pregnant women undergoing prenatal care covering issues related to breastfeeding, obtained satisfactory agreement among experts with an average content validity index of 0.89 ^(^ 30 ^)^, lower than that found in the current research. In this directive, research that developed and validated a Serious Game about expressing breast milk for occupational nurses who work in agro-industries, validated the usability of this game in the form of an application for such nurses, with an index of 83.89 on the SUS scale ^(^ 26 ^)^, a lower index than that found with the application now evaluated using the same scale. 

Regarding usability characteristics, a study on the “Diabetes in focus” application with nurses revealed a range of 87.75 to 90.75, indicating high ease of learning and generating satisfaction, achieving a classification of the best achievable ^(^ 24 ^)^, which is similar to the characteristics of the present investigation, which even achieved an even greater scope. 

Another evaluation investigation into a prototype mobile application on breastfeeding for healthcare professionals – the target audience for which the technology was intended – was also positively evaluated by participants, with the majority of evaluations classified as excellent or good ^(^ 31 ^)^. Internationally, in evaluating the usability and usefulness of a mobile application entitled “MoomMae”, developed in Thailand through quantitative and qualitative research, with a sample of 21 women, it also found a positive evaluation, demonstrating that the application has a great potential to be a useful self-management tool for breastfeeding mothers in Thailand ^(^ 28 ^)^. 

It is observed that the use of technologies, with emphasis on mobile devices, is expanding in the health area, with the purpose of assisting and expanding knowledge, being made available to both patients and professionals with the aim of informing, instruct, record, display, guide, remind, alert and/or communicate ^(^ 32 ^)^. However, for this type of technology to achieve these purposes, it is necessary to meet some crucial aspects that indicate the quality of software contained in the SUS scale, used in the present study, which covers a set of aspects of the system such as complexity, need for support, interface, among others. Therefore, this scale has a high level of validity for measuring the use of an application, as it is a robust and reliable assessment instrument ^(^ 23 ^)^, which classified the mobile application “ *Descomplicando a Amamentação*” as a device with the best usability achievable, which reflects in easy use and high user satisfaction. 

In this way, usability tests become increasingly essential before making an application available to the end user, configuring one of the main parameters to make a mobile application easy to be used by users, in addition to allowing them to achieve their specified objectives ^(^ 33 ^)^. Thus, based on the findings in this study, it is possible to infer that key points such as efficiency, effectiveness and satisfaction, which determine the user experience when involved with an application, were achieved. 

As a limitation of the research, it is pointed out that the study was carried out in a single context, since responses may be different between family members in the context of primary care or other hospital units in Brazil or, even, with a different level of education than the participants in the current investigation. Another possibility refers to the scarcity of publications on mobile applications evaluated by pregnant women, postpartum women and family members of newborns, which limits the comparison of results.

The data from this research should motivate health professionals and future professionals to evaluate and use technological means that are available in society, in order to promote health education. Furthermore, the use of educational health technologies, such as the one in question, is capable of motivating the target audience, through a technical and scientific basis, bringing knowledge, autonomy, safety and empowerment to the achievement, protection and duration of breastfeeding and, consequently, reducing neonatal morbidity and mortality.

## Conclusion

The “ *Descomplicando a Amamentação*” application was satisfactorily evaluated, presenting excellent ratings in the evaluation of semantics, appearance and usability by the target audience, indicating that this technology is understandable, relevant, relevant and easy to use, demonstrating excellent potential for use as educational health technology with families. It is currently available for free download for the Android operating system on the Google Play Store platform. 

The mobile application is an innovative technology in the health sector, as it was developed based on official recommendations and scientific literature, in addition to providing mothers and family members with the tools to incorporate good breastfeeding practices. It is expected that the research will motivate health professionals, including nurses and academics, regarding the use of technological productions in support of active learning methodologies in health.
